# Validity and Reproducibility of a Self-administered Food Frequency Questionnaire Used in the Baseline Survey of the JPHC Study Cohort I

**DOI:** 10.2188/jea.13.1sup_125

**Published:** 2007-11-30

**Authors:** Yoshitaka Tsubono, Minatsu Kobayashi, Satoshi Sasaki, Shoichiro Tsugane

**Affiliations:** 1 Epidemiology and Biostatistics Division, National Cancer Center Research Institute East.; 2 Division of Epidemiology, Department of Public Health and Forensic Medicine, Tohoku University Graduate School of Medicine.

**Keywords:** food frequency questionnaire, validity, reproducibility, dietary record, prospective study

## Abstract

We examined the validity and reproducibility of a 44-item food frequency questionnaire used in a baseline survey of the Japan Public Health Center-Based Prospective Study Cohort I conducted in February 1990. Subjects were 94 men and 107 women selected on a voluntary basis among respondents to the baseline survey. Four or five years after the baseline survey, they provided four 7-day diet records during a 1-year period, and then responded to the same questionnaire a second time. The median (range) for energy-adjusted correlation coefficients between 30 nutrients measured by the questionnaire and the diet records was 0.36 (0.06-0.81) for men and 0.37 (0.11-0.52) for women. The median correlation (range) for 17 food groups was 0.30 (0.08-0.75) for men and 0.28 (0.08-0.46) for women. The median correlation (range) for energy-adjusted correlation coefficients between the two questionnaires was 0.24 (0.04-0.69) for men and 0.50 (0.27-0.60) for women for the nutrients, and 0.34 (0.15-0.63) for men and 0.48 (0.18-0.55) for women for the food groups, respectively. The results indicate that this brief food frequency questionnaire provides reasonably valid and reproducible measures of consumption for many nutrients and food groups, and is useful for examining the association between diet and health in the Japanese population.

Epidemiologic studies of diet and chronic diseases commonly use a food frequency questionnaire (FFQ).^1^ A number of studies have examined the validity and the reproducibility of FFQs developed for Japanese populations.^2^^-^^8^ These questionnaires cover a relatively large number of food items, partly reflecting the large variety of food availability and consumption spectrum among the Japanese population. Although shorter questionnaires with fewer food items may be easier to complete and yield a higher response rate, only a few studies have assessed the validity and the reproducibility of brief FFQs for the Japanese population.

The objectives of this study are to assess the reproducibility and validity of a brief, 44-item food frequency questionnaire used in a baseline survey of the Japan Public Health Center-Based Prospective Study Cohort I. Part of the results have been reported elsewhere.^9^

## METHODS

Japan Public Health Center-based prospective study on cancer and cardiovascular diseases (JPHC Study)

The JPHC Cohort I was started in February 1990 when 61,595 men and women aged 40-59 years living in five Public Health Center Districts (Ninohe, Yokote, Saku, Katsushika-kita, and Ishikawa) were asked to complete a self-administered questionnaire including a 44-item food frequency section, which is referred to as the baseline food frequency questionnaire 1 (BFFQ1)^9^. Completed questionnaires were collected from 50,245 subjects, with a response rate of 82%.

### Subjects

The subjects of this validation study were a subsample of the participants in the prospective study in four districts, omitting one area in Tokyo (Katsushika-kita). We selected 122 men and 125 women on a voluntary basis. Table 1 summarizes the sequence of data collection. In three districts of mainland Japan (Ninohe, Yokote and Saku), we obtained 7-day diet records four times in different seasons in 1994, five years after the baseline survey, and administered the same questionnaire (BFFQ2) again in February, 1995. In one district of Okinawa Island, we collected 7-day diet records in the winter and summer of 1995, and the second questionnaire was administered in February 1996. We collected the diet records only twice in this sub-tropical area because the seasonal variations in diet were small. We also collected fasting blood and 24-hour stored urine samples twice, in February and August of 1994 (three mainland districts) or in 1995 (Okinawa district).

**Table 1.** The sequence of data collection for baseline food frequency questionnaires (BFFQs) and 7-day dietary records (DRs)

	Public Health Center Areas (Prefecture)			

Period	Ninohe (Iwate)	Yokote (Akita)	Saku (Nagano)	Ishikawa (Okinawa)
Feb, 1990^1^	BFFQ1	BFFQ1	BFFQ1	BFFQ1

Feb, 1994	DRI^2^	DRI^2^	DRI^2^	
May, 1994	DR^2^	DR^2^	DR^2^	
Aug, 1994	DR3^2^	DR3^2^	DR3^2^	
Nov, 1994	DR^4^	DR^4^	DR^4^	

Feb, 1995	BFFQ2	BFFQ2	BFFQ2	DRI^2^
Aug, 1995				DR2^2^

Feb, 1996				BFFQ2

^1^Baseline survey for the main cohort.

^2^Fasting blood and 24-hour stored urine were also collected.

Twenty men and 12 women who failed to provide their complete diet records were excluded, along with 8 men and 6 women who either did not respond or provide complete information for the BFFQ1 or BFFQ2. Subsequently, the remaining 94 men and 107 women were included in this analysis.

### Food Frequency Questionnaire

The food frequency questionnaire asked about the usual consumption of 44 food items during the previous month. Standard portions/units were neither specified nor asked. Four frequency categories were employed for the majority of foods (almost never / 1-2 days per week / 3-4 days per week / almost everyday). For rice and bean paste soup, inquiries were made as to the number of bowls consumed per day. Questions on the frequency of alcohol consumption covered six frequency categories (almost never / 1-3 days per month / 1-2 days per week / 3-4 days per week / 5-6 days per week / almost everyday), and queried those who consumed 1-2 days per week or more about the usual combinations and amounts of five beverages (sake, shochu, beer, whiskey, and other) consumed in a day, e.g., one bottle of beer and one bottle of sake. For 9 non-alcoholic beverages, six frequency categories were used (almost never / 1-2 days per week / 3-4 days per week / 1-2 cups per day / 3-4 cups per day / 5 or more cups per day).

Based on the dietary record data, we developed a food composition table that corresponded to the food items listed in the questionnaire, and determined standard portions/units for each food category using the observed median values. The food composition table was developed and standard portions/units were assigned separately for men and women. Daily nutrient intakes were calculated by multiplying the consumption frequency of each food by the nutrient content of the standard portions/units and summed these values for all foods. Calculation of total energy and 30 nutrients was done with the Standard Food Composition Table^11^ and other sources. Consumption of 17 food groups was also determined.

### Dietary Records

We collected weighed dietary records over seven consecutive days by a method used in the National Nutrition Survey^12^ with some modifications. Research dietitians instructed the subjects to record all foods and beverages prepared and consumed in a specially designed booklet. Participants were asked to provide detailed descriptions of each food, including the method of preparation and recipes whenever possible. The dietitians checked the records at the subjects^1^ homes during the survey and reviewed them in a standardized way after completion. The mean daily consumption of nutrients and food groups was calculated.

### Statistical Analysis

To assess the correspondence between DRs and BFFQ2, we compared the mean daily intakes and computed Spearman correlation coefficients for each nutrient and food group. For selected nutrients, we calculated Spearman correlation coefficients between serum and urinary levels (mean of the two measurements) and intakes measured by BFFQ2. To examine the 5-year reproducibility of the questionnaire, we compared the mean daily nutrient intakes and computed Spearman correlation coefficients between BFFQ1 and BFFQ2. The correlation coefficients were energy-adjusted by the residual method.^1^

## RESULTS

Table 2 shows the validity of BFFQ2 for nutrient intakes. The mean daily intakes calculated from BFFQ2 tended to be lower than those computed from DR. For men, energy-adjusted correlation coefficients between BFFQ2 and DR ranged from 0.06 (polyunsaturated fatty acid) through 0.81 (alcohol), with a median of 0.36. For women, it ranged from 0.11 (niacin) through 0.52 (alpha-carotene), with a median of 0.37. The correlations for fatty acids were generally higher when their respective consumption was expressed in terms of the percentage of total fatty acid intake.

**Table 2.** Energy, nutrient intake and Spearman rank correlation coefficients between nutrient intakes assessed with DR^1^ and those with BFFQ2^2^

	DR			BFFQ2			Spearman correlation		
		
	Mean	(± SD)	Median	Mean	(± SD)	Median	Crude	Energy-adjusted^3^	% of TFA
Men (n=94)									
Energy (kcal/day)	2334	(± 431)	2322	1993	(± 573)	2032	0.52		
Protein (g/day)	92.7	(± 15.9)	93.0	64.5	(± 18.2)	65.9	0.43	0.28	
Total fat (g/day)	59.2	(± 11.0)	61.2	34.7	(± 11)	34.7	0.18	0.30	
Carbohydrate (g/day)	314.4	(± 81.3)	306.1	307.9	(± 108)	305.3	0.61	0.51	
Alcohol (g/day)	22.3	(± 22.4)	15.3	23.4	(± 23.6)	17.9	0.79	0.81	
Calcium (mg/day)	614.6	(± 179.6)	611.7	550.3	(± 267.6)	547.1	0.56	0.56	
Phosphorus (mg/day)	1405.0	(± 272.8)	1392.2	1163.5	(± 361.1)	1162.0	0.58	0.56	
Iron (mg/day)	12.8	(± 2.6)	12.8	8.3	(± 2.4)	8.6	0.36	0.31	
Sodium (mg/day)	5333.9	(± 1272.3)	5248.5	2490.6	(± 1018.8)	2540.5	0.49	0.33	
Potassium (mg/day)	3188.7	(± 652.0)	3166.3	2229.5	(± 682.3)	2173.3	0.37	0.38	
Retinal (mg/day)	438.0	(± 480.3)	276.3	771.1	(± 716.8)	252.9	0.35	0.36	
Alpha carotene (*µ*g/day)	431.4	(± 257.6)	344.1	204.1	(± 176.6)	163.5	0.25	0.29	
Beta carotene (*µ*g/day)	2514.4	(± 995.0)	2289.4	1408.2	(± 615.6)	1271.2	0.29	0.26	
Vitamin B_1_ (mg/day)	1.30	(± 0.28)	1.31	0.91	(± 0.26)	0.96	0.35	0.36	
Vitamin B_2_ (mg/day)	1.55	(± 0.37)	1.49	1.37	(± 0.50)	1.34	0.45	0.43	
Niacin (mg/day)	21.8	(± 4.1)	21.9	13.7	(± 4.1)	13.8	0.25	0.14	
Vitamin C (mg/day)	126.6	(± 37.9)	125.5	85.5	(± 29.0)	80.7	0.33	0.38	
Selenium (mg/day)	182.7	(± 42.6)	184.4	99	(± 36.3)	95.7	0.45	0.39	
Cholesterol (mg/day)	418.7	(± 97.7)	408.1	281	(± 103)	278.1	0.35	0.36	
Lycopene (mg/day)	3011.1	(± 2808.0)	2107.6	3397	(± 11695.5)	590.3	0.12	0.08	
Water-soluble fiber (g/day)	2.09	(± 0.69)	2.05	1.20	(± 0.38)	1.15	0.29	0.35	
Water-insoluble fiber (g/day)	10.49	(± 2.66)	10.34	7.14	(± 2.14)	7.34	0.36	0.43	
Daidzein (mg/day)	15.7	(± 7.1)	14.6	11.3	(± 5.2)	11.9	0.53	0.51	
Genistein (mg/day)	25.2	(± 11.3)	23.6	17.9	(± 8.1)	18.1	0.53	0.51	
SFA (g/day)	16.00	(± 3.63)	16.31	12.76	(± 5.64)	12.43	0.32	0.42	0.20
MUFA (g/day)	19.58	(± 3.93)	19.58	10.62	(± 3.32)	10.60	0.13	0.23	0.36
PUFA (g/day)	15.83	(± 3.10)	15.64	6.69	(± 1.89)	6.89	0.06	0.06	0.31
n-3 PUFA (g/day)	3.48	(± 0.95)	3.42	1.29	(± 0.57)	1.26	0.33	0.20	0.38
n-6 PUFA (g/day)	12.27	(± 2.53)	12.02	5.34	(± 1.44)	5.62	0.08	0.14	0.30
C20:5(n3) (g/day)	0.56	(± 0.29)	0.52	0.03	(± 0.02)	0.03	0.55	0.39	0.51
C22:6(n3) (g/day)	0.13	(± 0.06)	0.12	0.04	(± 0.03)	0.04	0.52	0.36	0.51
Women (n=107)									
Energy (kcal/day)	1814	(± 322)	1796	1355	(± 331.9)	1333	0.38		
Protein (g/day)	76.2	(± 13.4)	76.1	52.3	(± 14)	51.0	0.33	0.34	
Total fat (g/day)	52.7	(± 9.8)	52.1	31.8	(± 10.5)	30.0	0.10	0.41	
Carbohydrate (g/day)	256.1	(± 58.6)	260.8	211.2	(± 51.7)	209.9	0.48	0.33	
Alcohol (g/day)	1.6	(± 2.9)	0.80	0.8	(± 3.9)	0.0	0.44	0.34	
Calcium (mg/day)	594.3	(± 165.2)	580.5	564.3	(± 272.1)	577.0	0.46	0.37	
Phosphorus (mg/day)	1167.7	(± 224.7)	1187.7	954.8	(± 301.7)	949.2	0.41	0.44	
Iron (mg/day)	11.2	(± 2.5)	11.2	7.2	(± 2.1)	7.2	0.38	0.30	
Sodium (mg/day)	4662.3	(± 1150.3)	4571.7	2064.0	(± 843)	2086.4	0.54	0.49	
Potassium (mg/day)	2926.8	(± 656.9)	2981.0	2101.8	(± 648.2)	2044.1	0.35	0.37	
Retinol (mg/day)	366.0	(± 430.0)	206.2	681.7	(± 786.4)	196.8	0.38	0.34	
Alpha carotene (*µ*g/day)	416.0	(± 212.3)	347.0	208.0	(± 118.1)	168.3	0.49	0.52	
Beta carotene (*µ*g/day)	2494.7	(± 995.4)	2302.5	1504.5	(± 583.3)	1416.9	0.41	0.38	
Vitamin B_1_ (mg/day)	1.11	(± 0.23)	1.10	0.76	(± 0.19)	0.74	0.32	0.22	
Vitamin B_2_ (mg/day)	1.37	(± 0.31)	1.39	1.29	(± 0.49)	1.22	0.36	0.39	
Niacin (mg/day)	16.9	(± 3.3)	16.5	10.2	(± 2.77)	9.9	0.27	0.11	
Vitamin C (mg/day)	134.6	(± 49.8)	126.5	96.9	(± 33.8)	91.8	0.35	0.29	
Selenium (mg/day)	148.0	(± 34.3)	145.9	79.8	(± 25)	75.8	0.43	0.45	
Cholesterol (mg/day)	359.1	(± 87.4)	357.3	253.7	(± 94.3)	250.7	0.26	0.30	
Lycopene (mg/day)	3239.8	(± 2581.7)	2449.5	2179.5	(± 4225.5)	720.8	0.03	0.11	
Water-soluble fiber (g/day)	2.11	(± 0.75)	2.00	1.33	(± 0.39)	1.29	0.47	0.42	
Water-insoluble fiber (g/day)	9.88	(± 2.84)	9.50	6.60	(± 1.74)	6.55	0.49	0.43	
Daidzein (mg/day)	13.5	(± 6.0)	13.0	10.6	(± 4.5)	11.5	0.55	0.49	
Genistein (mg/day)	21.7	(± 9.4)	20.9	16.9	(± 7.1)	17.5	0.54	0.48	
SFA (g/day)	14.78	(± 3.47)	14.52	12.24	(± 5.70)	12.30	0.26	0.50	0.48
MUFA (g/day)	17.40	(± 3.56)	16.89	9.62	(± 3.11)	9.33	0.08	0.39	0.37
PUFA (g/day)	13.82	(± 2.61)	13.42	5.81	(± 1.54)	5.66	0.15	0.22	0.49
n-3 PUFA (g/day)	2.99	(± 0.74)	2.89	1.16	(± 0.44)	1.09	0.41	0.35	0.50
n-6 PUFA (g/day)	10.77	(± 2.11)	10.57	4.61	(± 1.20)	4.59	0.09	0.15	0.35
C20:5(n3) (g/day)	0.45	(± 0.20)	0.44	0.02	(± 0.01)	0.02	0.54	0.42	0.50
C22:6(n3) (g/day)	0.11	(± 0.04)	0.11	0.02	(± 0.02)	0.03	0.48	0.38	0.50

^1^DR, dietary records.

^2^BFFQ2, Baseline food frequency questionnaire. in 1995 (for Ishikawa, in 1996)

^3^Energy was adjusted by residual method.

For n=94, r>0.21 = p<0.05, r>0.27 = p<0.01, r>0.34 = p<0.001.

For n=107, r>0.19 = p<0.05, r>0.25 = p<0.01, r>0.32 = p<0.001.

Table 3 shows the validity of BFFQ2 for consumption of food groups. The mean daily intakes calculated from BFFQ2 tended to be lower than those computed from DR. For men, energy-adjusted correlation coefficients between BFFQ2 and DR ranged from 0.08 (seasonings and spices) through 0.75 (alcoholic beverages), with a median of 0.30. For women, it ranged from 0.08 (seasonings and spices) through 0.46 (milk and dairy products), with a median of 0.28.

**Table 3.** Food group intakes (g/day) assessed with DR for 28 or 14 days and BFFQ2 in 4 areas and their correlations

Sex	DR			BFFQ2^1^			Spearman correlation	
			
Food group	Mean	(± SD)	Median	Mean	(± SD)	Median	Crude	Energy-adjusted^2^
Men (n=94)								
Cereals	311.1	(± 86.9)	294.0	342.7	(± 131.9)	326.3	0.59	0.36
Potatoes and starches	48.6	(± 20.3)	46.8	19.5	(± 12.9)	11.5	0.13	0.24
Fats and oils	11.3	(± 4.5)	10.3	1.0	(± 1.5)	1.2	0.18	0.17
Pulses	86.9	(± 36.3)	81.6	61.2	(± 27.5)	62.1	0.43	0.39
Fish and shellfish	137.1	(± 46.1)	137.6	48.4	(± 32.8)	40.0	0.49	0.37
Meats	76.4	(± 32.4)	69.2	41.9	(± 19.8)	42.4	0.10	0.18
Eggs	39.3	(± 13.8)	37.5	30.8	(± 15.7)	25.0	0.24	0.25
Milk and dairy products	122.1	(± 109.4)	111.7	249.6	(± 227.9)	300.0	0.52	0.46
Vegetables	309.7	(± 88.1)	305.9	184.1	(± 127.7)	160.9	0.26	0.27
Green and yellow	106.1	(± 47.2)	99.2	45.9	(± 21.0)	42.5	0.24	0.25
Pickled	34.4	(± 30.0)	25.2	24.2	(± 19.2)	22.5	0.66	0.59
Fruits	121.2	(± 87.0)	93.4	93.1	(± 54.1)	105.3	0.52	0.55
Fungi	8.6	(± 6.2)	7.4	3.0	(± 2.3)	2.1	0.33	0.30
Algae	7.8	(± 6.3)	5.8	2.0	(± 1.0)	1.9	0.25	0.21
Alcoholic beverages	311.9	(± 282.5)	254.3	287.5	(± 322.0)	180.0	0.73	0.75
Non-alcoholic beverages	268.9	(± 137.5)	250.7	636.7	(± 315.3)	639.6	0.50	0.54
Seasonings and spices	35.9	(± 10.5)	34.6	2.4	(± 1.9)	2.0	0.08	0.08
Women (n=107)								
Cereals	220.4	(± 49.9)	217.4	216.687	(± 56.2)	213	0.46	0.27
Potatoes and starches	48.1	(± 22.3)	44.8	21.079	(± 12.7)	11.342	0.21	0.19
Fats and oils	9.7	(± 3.6)	9.2	1.104	(± 1.4)	1.07	0.11	0.16
Pulses	74.1	(± 28.6)	71.0	57.9	(± 24.4)	62.6	0.42	0.43
Fish and shellfish	107.5	(± 32.9)	102.6	39.7	(± 25.9)	31.9	0.45	0.32
Meats	58.9	(± 28.4)	52.1	33.3	(± 15.4)	31.7	0.19	0.26
Eggs	33.6	(± 12.2)	32.6	28.9	(± 16.0)	24.8	0.28	0.28
Milk and dairy products	149.7	(± 93.0)	154.7	291.5	(± 247.5)	370.0	0.59	0.46
Vegetables	297.6	(± 90.9)	287.1	176.3	(± 78.1)	167.1	0.36	0.31
Green and yellow	109.3	(± 46.9)	99.7	54.1	(± 23.9)	45.5	0.29	0.19
Pickled	30.5	(± 26.1)	22.6	24.0	(± 16.1)	20.0	0.61	0.44
Fruits	156.3	(± 83.9)	141.8	139.8	(± 104.4)	150.0	0.45	0.35
Fungi	7.9	(± 5.4)	6.7	3.5	(± 2.5)	2.1	0.27	0.28
Algae	7.1	(± 5.5)	5.4	1.7	(± 0.9)	1.6	0.14	0.19
Alcoholic beverages	22.9	(± 40.5)	7.9	12.9	(± 51.8)	0.0	0.44	0.40
Non-alcoholic beverages	275.7	(± 114.0)	266.9	623.3	(± 280.7)	625.7	0.40	0.39
Seasonings and spices	32.9	(± 9.9)	33.1	2.4	(± 1.5)	1.9	0.12	0.08

^1^BFFQ2, Baseline food frequency questionnaire. in 1995 (for Ishikawa, in 1996)

^2^Energy was adjusted by residual method.

For n=94, r>0.21 = p<0.05, r>0.27 = p<0.01, r>0.34 = p<0.001.

For n=107, r>0.19 = p<0.05, r>0.25 = p<0.01, r>0.32 = p<0.001.

Table 4 presents the validity of BFFQ2 for food intakes (g/day) and food frequency (times/day). The validity for food intakes (g/day) and food frequency (times/day) was comparable, although the correlation coefficients were different with the kind of food.

**Table 4.** Spearman correlation between BFFQ2^1^ and food intakes (g/day) or food frequency (times/day) assessed with DR for 28 or 14 days

Food	Men (n=94)		Women (n=107)	
	
	(g/day)	(times/day)	(g/day)	(times/day)
Rice	0.74	0.46	0.66	0.63
Miso	0.41	0.50	0.49	0.54
Noodle	0.29	0.24	0.50	0.42
Instant noodle	0.29	0.28	0.20	0.20
Bread	0.53	0.57	0.58	0.60
Butter	0.40	0.40	0.34	0.28
Fruits	0.52	0.59	0.41	0.44
Green vegetable	0.30	0.19	0.21	0.14
Yellow vegetable	0.30	0.48	0.23	0.48
Other vegetable	0.01	0.18	0.14	0.21
Dressing	0.14	0.20	0.27	0.33
Pickled vegetable	0.67	0.64	0.61	0.63
Mushroom	0.30	0.37	0.27	0.41
Potatoes	0.12	0.47	0.21	0.36
Seaweed	0.25	0.37	0.14	0.33
Soy	0.41	0.55	0.34	0.49
Other beans	0.13	0.21	0.11	0.13
Egg	0.20	0.14	0.28	0.30
Dairy	0.47	0.30	0.66	0.60
Cheese	0.41	0.40	0.43	0.45
Beef	0.25	0.30	0.42	0.40
Pork	0.18	0.18	0.30	0.26
Chicken	0.23	0.31	0.23	0.22
Bacon	0.29	0.22	0.29	0.29
Liver	0.20	0.17	0.29	0.26
Fish	0.27	0.07	0.20	0.00
Dry fish	0.55	0.47	0.33	0.25
Fish egg	0.61	0.56	0.47	0.48
Shiokara (salt-cured preserve)	0.43	0.43	0.34	0.34
Japanese tea	0.57	0.57	0.63	0.63
Chinese tea	0.22	0.23	0.34	0.34
Black tea	0.05	0.05	0.35	0.35
Other tea	0.05	0.04	0.23	0.22
Coffee	0.42	0.42	0.38	0.38
Milk	0.51	0.45	0.65	0.60
Juice	0.28	0.27	0.26	0.24
Fruit juice	0.17	0.17	0.17	0.18
Vegetable juice	0.17	0.18	0.17	0.18
Sugar	0.16	0.02	0.11	0.19

^1^BFFQ2, Baseline food frequency questionnaire in 1995 (for Ishikawa, in 1996).

For n=94, r>0.21 = p<0.05, r>0.27 = p<0.01, r>0.34 = p<0.001.

For n=107, r>0.19 = p<0.05, r>0.25 = p<0.01, r>0.32 = p<0.001.

Table 5 indicates the validity of BFFQ2 using biomarkers of selected nutrients as the reference standard. Among men, dietary intake measured by the questionnaire showed a moderate correlation with serum levels of n-3 polyunsaturated fatty acid, eicosapentaenoic acid, docosahexanoic acid as a percentage of total fatty acid, serum levels of alpha-carotene, beta-carotene, daidzein, genistein, and urinary levels of sodium. Among women, correlations between the questionnaire estimates and biomarkers were generally low.

**Table 5.** Spearman rank correlation coefficients between nutrient intakes assessed with BFFQ2^1^ and corresponding biomarker

Biomarker		Men				Women		
	
		n	Crude	Energy-adjusted**	% of TFA	n	Crude	Energy-adjusted^2^
Serum phospholipid								
SFA	% of TFA	83	0.11	0.07	0.06	-	-	-
MUFA	% of TFA	83	-0.13	-0.08	0.32	-	-	-
PUFA	% of TFA	83	-0.05	-0.01	0.03	-	-	-
n-3 PUFA	% of TFA	83	0.48	0.31	0.49	-	-	-
n-6 PUFA	% of TFA	83	-0.34	-0.01	-0.17	-	-	-
C20:5(n3)	% of TFA	83	0.54	0.41	0.44	-	-	-
C22:6(n3)	% of TFA	83	0.41	0.30	0.42	-	-	-
Serum								
alpha-carotene	mg/ml	82	0.42	0.48	-	96	0.18	0.28
beta-carotene	mg/ml	82	0.41	0.42	-	96	-0.03	-0.03
Lycopene	mg/ml	82	0.23	0.18	-	96	0.10	0.11
Selenium	*µ*mol/L	80	-0.04	0.08	-	89	-0.28	-0.14
Daidzein	nmol/L	87	0.39	0.34	-	104	0.17	0.10
Genistein	nmol/L	87	0.38	0.30	-	104	0.24	0.15
Plasma								
ascorbic acid	mg/dl	82	-0.08	-0.13	-	95	0.02	-0.08
Erythrocytes								
Selenium	nmol/g	84	-0.09	0.07	-	96	-0.20	0.03
Urine								
sodium	mmol/day	32	0.38	0.34	-	57	0.12	0.15
potassium	mmol/day	32	0.21	0.26	-	57	-0.02	0.05
Daidzein	*µ*mol/day	32	0.19	0.13	-	57	-0.01	0.08
Genistein	*µ*mol/day	32	0.12	0.05	-	57	-0.04	-0.03

^1^BFFQ2, Baseline food frequency questionnaire in 1995 (for Ishikawa, in 1996).

^2^Energy was adjusted by residual method.

For n=96 or n=95, r>0.21 = p<0.05, r>0.27 = p<0.01, r>0.34 = p<0.001.

For n=82 or n=83, r>0.22 = p<0.05, r>0.29 = p<0.01, r>0.36 = p<0.001.

For n=57, r>0.27 = p<0.05, r>0.34 = p<0.01, r>0.43 = p<0.001.

For n=32, r>0.35 = p<0.05, r>0.45 = p<0.01, r>0.56 = p<0.001.

Table 6 shows the 5-year reproducibility of the questionnaire for nutrient intakes. The mean daily intakes were generally comparable between BFFQ1 and BFFQ2. For men, energy-adjusted correlation coefficients between BFFQ1 and BFFQ2 ranged from 0.04 (lycopene) through 0.69 (alcohol), with a median of 0.24. For women, it ranged from 0.27 (lycopene) through 0.60 (total fat), with a median of 0.50.

**Table 6.** Energy, nutrient intake and Spearman rank correlation coefficients between nutrient intakes assssed with BFFQ1^1^ and BFFQ2^2^

	BFFQ1			BFFQ2			Spearman correlation		
		
	Mean	(± SD)	Median	Mean	(± SD)	Median	Crude	Energy-adjusted^3^	% of TFA
Men(n=94)									
Energy (kcal/day)	1866	(± 501)	1798	1993	(± 573)	2032	0.49		
Protein (g/day)	65.2	(± 17.2)	64.1	64.5	(± 18.2)	65.9	0.53	0.18	
Total fat (g/day)	34.4	(± 11.3)	34.1	34.7	(± 11)	34.7	0.38	0.18	
Carbohydrate (g/day)	315.3	(± 94.5)	302.8	307.9	(± 108)	305.3	0.49	0.38	
Alcohol (g/day)	23.4	(± 26.0)	17.9	23.4	(± 23.6)	17.9	0.78	0.69	
Calcium (mg/day)	534.3	(± 285.4)	510.9	550.3	(± 267.6)	547.1	0.52	0.33	
Phosphorus (mg/day)	1157.6	(± 354.4)	1144.9	1163.5	(± 361.1）	1162.0	0.58	0.19	
Iron (mg/day)	8.4	(± 2.2)	8.7	8.3	(± 2.4)	8.6	0.51	0.11	
Sodium (mg/day)	2519.2	(± 1089.9)	2575.8	2490.6	(± 1018.8)	2540.5	0.53	0.30	
Potassium (mg/day)	2139.1	(± 625.8)	2204.6	2229.5	(± 682.3)	2173.3	0.45	0.15	
Retinol (mg/day)	696.6	(± 740.0)	213.3	771.1	(± 716.8)	252.9	0.10	0.06	
Alpha carotene (*µ*g/day)	159.1	(± 121.3)	128.9	204.1	(± 176.6)	163.5	0.41	0.39	
Beta carotene (*µ*g/day)	1259.6	(± 550.3)	1216.5	1408.2	(± 615.6)	1271.2	0.35	0.34	
Vitamin B_1_ (mg/day)	0.92	(± 0.25)	0.95	0.91	(± 0.26)	0.96	0.57	0.20	
Vitamin B_2_ (mg/day)	1.30	(± 0.47)	1.26	1.37	(± 0.50)	1.34	0.39	0.14	
Niacin (mg/day)	13.0	(± 3.5)	13.2	13.7	(± 4.1)	13.8	0.44	0.25	
Vitamin C (mg/day)	82.1	(± 26.3)	81.3	85.5	(± 29.0)	80.7	0.34	0.24	
Selenium (mg/day)	99.7	(± 30.8)	93.9	99.2	(± 36.3)	95.7	0.47	0.32	
Cholesterol (mg/day)	273	(± 92)	273	281	(± 103)	278	0.22	0.12	
Lycopene (mg/day)	2064.1	(± 5099.9)	429.7	3397	(± 11695.5)	590.3	0.29	0.04	
Water-soluble fiber (g/day)	1.15	(± 0.35)	1.13	1.20	(± 0.38)	1.15	0.40	0.20	
Water-insoluble fiber (g/day)	7.15	(± 1.92)	7.50	7.14	(± 2.14)	7.34	0.55	0.26	
Daidzein (mg/day)	11.5	(± 5.5)	10.9	11.3	(± 5.2)	11.9	0.66	0.49	
Genistein (mg/day)	18.1	(± 8.6)	18.1	17.9	(± 8.1)	18.1	0.65	0.50	
SFA (g/day)	12.57	(± 6.20)	11.73	12.76	(± 5.64)	12.43	0.43	0.27	0.50
MUFA (g/day)	10.52	(± 3.51)	10.40	10.62	(± 3.32)	10.60	0.34	0.13	0.50
PUFA (g/day)	6.76	(± 1.93)	6.92	6.69	(± 1.89)	6.89	0.49	0.20	0.47
n-3 PUFA (g/day)	1.33	(± 0.53)	1.30	1.29	(± 0.57)	1.26	0.45	0.30	0.38
n-6 PUFA (g/day)	5.37	(± 1.49)	5.48	5.34	(± 1.44)	5.62	0.47	0.15	0.44
C20:5(n3) (g/day)	0.03	(± 0.02)	0.03	0.03	(± 0.02)	0.03	0.64	0.49	0.49
C22:6(n3) (g/day)	0.0022	(± 0.0017)	0.0017	0.04	(± 0.03)	0.04	0.60	0.46	0.48
Women (n=107)									
Energy (kcal/day)	1399	(± 366)	1403	1355	(± 331.9)	1333	0.58		
Protein (g/day)	54.4	(± 15.5)	55.4	52.3	(± 14)	51.0	0.65	0.58	
Total fat (g/day)	32.8	(± 11.3)	32.4	31.8	(± 10.5)	30.0	0.51	0.60	
Carbohydrate (g/day)	218.8	(± 59.1)	218.2	211.2	(± 51.7)	209.9	0.61	0.51	
Alcohol (g/day)	0.8	(± 5.6)	0.0	0.8	(± 3.9)	0.0	0.66	0.50	
Calcium (mg/day)	537.6	(± 279.0)	484.4	564.3	(± 272.1)	577.0	0.62	0.49	
Phosphorus (mg/day)	963.1	(± 323.8)	973.5	954.8	(± 301.7)	949.2	0.64	0.51	
Iron (mg/day)	7.6	(± 2.0)	7.9	7.2	(± 2.1)	7.2	0.65	0.38	
Sodium (mg/day)	2151.4	(± 951.8)	2223.2	2064.0	(± 843)	2086.4	0.65	0.58	
Potassium (mg/day)	2090.7	(± 636.8)	2058.1	2101.8	(± 648.2)	2044.1	0.64	0.50	
Retinol (mg/day)	751.5	(± 839.9)	206.2	681.7	(± 786.4)	196.8	0.49	0.36	
Alpha carotene (*µ*g/day)	192.0	(± 99.3)	163.9	208.0	(± 118.1)	168.3	0.62	0.52	
Beta carotene (*µ*g/day)	1517.6	(± 496.7)	1510.8	1504.5	(± 583.3)	1416.9	0.55	0.43	
Vitamin B_1_ (mg/day)	0.79	(± 0.22)	0.81	0.76	(± 0.19)	0.74	0.65	0.45	
Vitamin B_2_ (mg/day)	1.28	(± 0.50)	1.26	1.29	(± 0.49)	1.22	0.60	0.53	
Niacin (mg/day)	10.6	(± 3.0)	10.5	10.2	(± 2.77)	9.9	0.66	0.42	
Vitamin C (mg/day)	95.9	(± 29.7)	96.2	96.9	(± 33.8)	91.8	0.48	0.39	
Selenium (mg/day)	84.8	(± 27.4)	83.5	79.8	(± 25)	75.8	0.69	0.56	
Cholesterol (mg/day)	275	(± 94)	271	254	(± 94)	251	0.43	0.46	
Lycopene (mg/day)	1010.8	(± 1403.1)	548.7	2179.5	(± 4225.5)	720.8	0.47	0.27	
Water-soluble fiber (g/day)	1.38	(± 0.38)	1.4	1.33	(± 0.39)	1.29	0.56	0.48	
Water-insoluble fiber (g/day)	6.99	(± 1.75)	7.0	6.60	(± 1.74)	6.55	0.61	0.49	
Daidzein (mg/day)	11.5	(± 4.6)	11.5	10.6	(± 4.5)	11.5	0.61	0.60	
Genistein (mg/day)	18.2	(± 7.1)	17.5	16.9	(± 7.1)	17.5	0.61	0.58	
SFA (g/day)	11.90	(± 5.98)	11.41	12.24	(± 5.70)	12.30	0.50	0.51	0.44
MUFA (g/day)	10.06	(± 3.55)	9.59	9.62	(± 3.11)	9.33	0.49	0.60	0.52
PUFA (g/day)	6.37	(± 1.77)	6.63	5.81	(± 1.54)	5.66	0.61	0.50	0.52
n-3 PUFA (g/day)	1.28	(± 0.51)	1.28	1.16	(± 0.44)	1.09	0.71	0.55	0.48
n-6 PUFA (g/day)	5.04	(± 1.34)	5.25	4.61	(± 1.20)	4.59	0.53	0.42	0.50
C20:5(n3) (g/day)	0.02	(± 0.011)	0.02	0.02	(± 0.01)	0.02	0.63	0.48	0.48
C22:6(n3) (g/day)	0.001	(± 0.001)	0.001	0.02	(± 0.02)	0.03	0.62	0.49	0.47

^1^BBQ1, Baseline food frequency questionnaire in 1990.

^2^BFFQ2, Baseline food frequency questionnaire in 1995 (for Ishikawa, in 1996).

^3^Energy was adjusted by residual method.

For n=94, r>0.21 = p<0.05, r>0.27 = p<0.01、r>0.34 = p<0.001.

For n=107, r>0.19 = p<0.05, r>0.25 = p<0.01, r>0.32 = p<0.001.

Table 7 shows the 5-year reproducibility of the questionnaire for consumption of a food group. The mean daily intakes were generally comparable between BFFQ1 and BFFQ2. For men, energy-adjusted correlation coefficients between BFFQ1 and BFFQ2 ranged from 0.15 (algae) through 0.63 (alcoholic beverages), with s median of 0.34. For women, it ranged from 0.18 (algae) through 0.55 (non-alcoholic beverages), with a median of 0.48.

**Table 7.** Food group intakes (g/day) and Spearman rank correlation between food group intakes assessed with BFFQ1^1^ and BFFQ2^2^

Sex	BFFQ1			BFFQ2			Spearman correlation	
			
Food group	Mean	(± SD)	Median	Mean	(± SD)	Median	Crude	Energy-adjusted^3^
Men (n=94)								
Cereals	365.9	(± 118.7)	362.6	342.7	(± 131.9)	326.3	0.36	0.22
Potatoes and starches	16.6	(± 13.1)	11.5	19.5	(± 12.9)	11.5	0.35	0.29
Fats and oils	0.9	(± 1.4)	0.0	1.0	(± 1.5)	1.2	0.55	0.52
Pulses	62.1	(± 28.6)	61.4	61.2	(± 27.5)	62.1	0.63	0.46
Fish and shellfish	53.2	(± 31.1)	50.0	48.4	(± 32.8)	40.0	0.50	0.35
Meats	41.1	(± 18.0)	38.1	41.9	(± 19.8)	42.4	0.37	0.37
Eggs	29.1	(± 15.3)	25.0	30.8	(± 15.7)	25.0	0.33	0.29
Milk and dairy products	244.0	(± 260.0)	136.6	249.6	(± 227.9)	300.0	0.50	0.36
Vegetables	169.1	(± 88.7)	160.2	184.1	(± 127.7)	160.9	0.37	0.29
Green and yellow	40.8	(± 21.0)	42.5	45.9	(± 21.0)	42.5	0.35	0.36
Pickled^4^	25.3	(± 19.0)	22.5	24.2	(± 19.2)	22.5	0.53	0.42
Fruits	83.1	(± 52.7)	69.6	93.1	(± 54.1)	105.3	0.40	0.32
Fungi	2.1	(± 1.6)	2.1	3.0	(± 2.3)	2.1	0.31	0.21
Algae	1.8	(± 1.1)	1.9	2.0	(± 1.0)	1.9	0.22	0.15
Alcoholic beverages	276.4	(± 327.6)	177.0	287.5	(± 322.0)	180.0	0.69	0.63
Non-alcoholic beverages	686.2	(± 338.4)	720.0	636.7	(± 315.3)	639.6	0.27	0.34
Seasonings and spices	2.3	(± 2.0)	2.0	2.4	(± 1.9)	2.0	0.16	0.18
Women (n=107)								
Cereals	229.6	(± 63.0)	223.7	216.687	(± 56.2)	213	0.55	0.48
Potatoes and starches	22.2	(± 13.5)	26.5	21.079	(± 12.7)	11.342	0.44	0.29
Fats and oils	1.3	(± 1.4)	1.1	1.104	(± 1.4)	1.07	0.43	0.41
Pulses	63.4	(± 24.3)	65.0	57.9	(± 24.4)	62.6	0.59	0.55
Fish and shellfish	42.4	(± 27.1)	43.0	39.7	(± 25.9)	31.9	0.67	0.48
Meats	38.0	(± 20.4)	32.4	33.3	(± 15.4)	31.7	0.51	0.49
Eggs	32.5	(± 14.9)	24.8	28.9	(± 16)	24.8	0.36	0.40
Milk and dairy products	254.1	(± 249.5)	174.3	291.5	(± 247.5)	370.0	0.53	0.48
Vegetables	175.9	(± 61.8)	171.2	176.3	(± 78.1)	167.1	0.50	0.39
Green and yellow	56.1	(± 22.1)	47.0	54.1	(± 23.9)	45.5	0.56	0.48
Pickled^4^	24.6	(± 16.7)	40.0	24.0	(± 16.1)	20.0	0.58	0.51
Fruits	125.9	(± 78.1)	150.0	139.8	(± 104.4)	150.0	0.44	0.32
Fungi	3.0	(± 2.5)	2.1	3.5	(± 2.5)	2.1	0.46	0.42
Algae	1.8	(± 0.9)	1.6	1.7	(± 0.9)	1.6	0.28	0.18
Alcoholic beverages	14.1	(± 99.9)	0.0	12.9	(± 51.8)	0.0	0.66	0.50
Non-alcoholic beverages	618.5	(± 328.1)	593.5	623.3	(± 280.7)	625.7	0.54	0.55
Seasonings and spices	3.0	(± 2.2)	1.9	2.4	(± 1.5)	1.9	0.43	0.36

^1^BFFQ1, Baseline food frequency questionnaire. in 1990

^2^BFFQ2, Baseline food frequency questionnaire in 1995 (for Ishikawa, in 1996)

^3^Energy was adjusted by residual method.

^4^Pickled plum (umeboshi) was included in pickled vegetables, and not in total vegetables but rather in fruits.

For n=94, r>0.21 = p<0.05, r>0.27 = p<0.01, r>0.34 = p<0.001.

For n=107, r>0.19 = p<0.05, r>0.25 = p<0.01, r>0.32 = p<0.001.

## DISCUSSION

We found that the mean daily intakes were lower in the questionnaire than in the diet records for most nutrients and food groups. This is probably due to the relatively small number of food items listed in the questionnaire (44 items). Thus, the questionnaire would underestimate the absolute levels of consumption of nutrients and food groups.

Correlations between the questionnaire and the dietary records were lower than those reported in Western studies,^1^ but comparable with those found in Japanese studies.^2^^-^^8^ The results indicate that the questionnaire is reasonably valid in ranking subjects and classifying them into quintiles according to the consumption of many nutrients and food groups.

Reproducibility of our questionnaire tended to be lower than reported in previous Western^1^ and Japanese^2^^-^^8^ studies. We administered the two questionnaires at a 5-year interval, which is substantially longer than the intervals in other studies (typically one year). The longer interval of questionnaire administration would result in the observed lower correlations. The reproducibility of the questionnaire in the present study was generally higher in women than in men, suggesting that patterns of nutrient and food consumption are more stable over time in women than in men among the Japanese population.

In conclusion, this brief, 44-item food frequency questionnaire used in the baseline survey of the Japan Public Health Center-Based Prospective Study Cohort I provides reasonably useful measures of intake for many nutrients and food groups. The questionnaire would be useful in examining the association between diet and health in the Japanese population.

## References

[1] Willett WC. Nutritional Epidemiology (2nd ed.). Oxford: Oxford University Press; 1998.

[2] Wakai K, Egami I, Kato K, Lin Y, Kawamura T, Tamakoshi A, . A simple food frequency questionnaire for Japanese diet - Part I, Development of questionnaire, and reproducibility and validity for food groups. J Epidemiol 1999;9:216-26. 10510578 10.2188/jea.9.216

[3] Egami I, Wakai K, Kato K, Lin Y, Kawamura T, Tamakoshi A, . A simple food frequency questionnaire for Japanese diet - Part II. Reproducibility and validity for nutrient intakes. J Epidemiol 1999;9:227-34. 10510579 10.2188/jea.9.227

[4] Tokudome S, Imaeda N, Tokudome Y, Fujiwara N, Nagaya T, Sato J, . Relative validity of a semi-quantitative food frequency questionnaire versus 28-day weighed diet records in Japanese female dietitians. Eur J Clin Nutr 2001;55:735-42. 11528486 10.1038/sj.ejcn.1601215

[5] Tsubono Y, Suzuki K, Watanabe Y, Nishino Y, Tsuji I, Watanabe T, . Food Frequency Questionnaire as a Screening Test. Nutr Cancer 2001;39:78-84. 11588906 10.1207/S15327914nc391_11

[6] Shimizu H, Ohwaki A, Kurisu Y, Takatsuka N, Ido M, Kawakami N, . Validity and reproducibility of a quantitative food frequency questionnaire for a cohort study in Japan. Jpn J Clin Oncol 1999;29:38-44. 10073150 10.1093/jjco/29.1.38

[7] Date C, Yamaguchi M, Tanaka H. Development of a food frequency questionnaire in Japan. J Epidemiol 1997;6(suppl):131S-136S. 10.2188/jea.6.3sup_131 8800285

[8] Sasaki S, Yanagibori R, Amano K. Self-administered diet history questionnaire on development of health education: a relative validation of the test-version by comparison with 3-day diet record in women. J Epidemiol 1998;8:203-15. 9816812 10.2188/jea.8.203

[9] Tsubono Y, Sasaki S, Kobayashi M, Akabane M, Tsugane S. Food composition and empirical weight methods in predicting nutrient intakes from food frequency questionnaire. Ann Epidemiol 2001;11:213-8. 11248586 10.1016/s1047-2797(00)00215-5

[10] Tsugane S, Fahey MT, Sasaki S, Baba S for JPHC Study Group. Alcohol consumption and all-cause and cancer mortality among middle-aged Japanese men: seven-year follow-up of the JPHC Study Cohort I. Am J Epidemiol 1999;50:1201-7. 10.1093/oxfordjournals.aje.a009946 10588080

[11] Science and Technology Agency of Japan. Standard Tables of Food Consumption in Japan, 4th revised ed. Tokyo: Printing Bureau, Ministry of Finance; 1982. (In Japanese)

[12] Ministry of Health and Welfare of Japan. Report of the National Nutrition Survey in 1994. Tokyo: Daiichi-Shuppan; 1996. (In Japanese)

